# Abstracts and Gleanings

**Published:** 1880-12-20

**Authors:** 


**Prolapsus Ani in Children.**—Dr. Basevi (Giornale Internazionalle delle Science Mediche) employs the following treatment in chronic cases: He first cauterizes slightly the protruding portion with nitrate of silver, and then reduces it, administering afterward, with the view of checking any tendency to enteritis, an enema of tannic acid, alum, and ice-cold water. Should this treatment prove insufficient, the child is placed on a bed with the nates upward, and steadied by two assistants, one of whom fixes the upper part of the body, while the other holds the knees elevated and somewhat abducted. The prolapsus having been reduced, the nates are brought together, and two strips of diachylon plaster, each about two inches wide, are passed from one trochanter to the other in as close proximity as possible to the perineum. To keep them in place, a spica bandage is applied around the lower portion of the body, and a piece ot gutta percha is added to protect the plaster from the contact of fecal matter. It is said that the apparatus can be left in position for two weeks. Cases of prolapsus ani in children that have, from want of attention, become chronic, are very unsatisfactory as far as treatment is concerned, and those physicians who expect a cure of such by the application of any one of the numerous astringent washes, or even of nitrate of silver, or any of the acids, will be disappointed in the majority of cases, unless strict attention is paid to keeping the child absolutely quiet, and the parts at rest.

It strikes us that the plan recommended by Dr. Basevi is a good one, and that the advantages to be gained from it are many.—*J. M. M. in Medical Herald.*

**Microscopical Examination of Vaccine Virus.**—We find in the "Proceedings of the Medical Society of the County of Kings" an interesting report by the committee on Hygiene, on the "causes of alleged futill and bad results following vaccination." Dr. N. B. Sizer, of the committee, gives the following results from the microscopical examination of vaccine virus; 58 specimens were examined:

| | |
|---|---|
| 20 | Fresh Lymph. |
| 1 | Cone. |
| 15 | Ivory slips, charged by Dr. S. |
| 20 | Ivory slips, commercial charging. |
| 2 | Crusts. |
| 58 | |

In twenty specimens of fresh lymph only three contained the shining micrococci, (so-called vaccine corpuscle). In eleven out of the twenty, despite care, red blood corpuscles were detected, absent *only* when the *spontaneously exuding* lymph alone was used. Any attempt to increase the yield by pressure or manipulation being followed by slight hemorrhage, perhaps microscopic only. Of the 15 slips charged by Dr. Sizer, Nos. 1 to 5, inclusive, charged with freely exuding lymph, contained no red blood cells. Nos. 6 to 15, where slight pressure was used, *all showed red blood cells.* Twenty slips, from various sources, showed eight with red blood cells, one *a humanized slip,* contained blood enough to show a pink color.

The cone was composed largely of *debris*. The following conclusions were reached:

1. The presence of blood in vaccine virus is easily detected by the microscope.

2. Blood is a frequent contamination of commercial lymph in a dried state.

3. Its presence is probably due to an effort at obtaining as large a portion of lymph as possible.

4. Its presence is highly dangerous in humanized lymph, but probably of no account in that from the calf.

5. The "so-called vaccine corpuscle" has not been found in quantity or at times sufficient to render it undoubtedly a specific appearance due to vaccine. It appears to be merely a microccoccus.

6. Vaccine cones as now offered in the market are undesirable forms of transmitting lymph.

Crusts which were examined consist largely of necrosed tissue. Probably only the thin, transparent to player, is usable.—*J. B. M., in Medical Herald.*

**Pneumatic Apparatus in Chronic Lung Diseases.**—When we consider that this method has been in vogue for only two years, its universal acceptance, and that from ten to fifteen per cent. of the entire population of the earth is carried away by chronic lung affections which prove so unmanageable, it is surprising that it was not adopted years ago. Different climates to suit different conditions of pulmonary diseases are urged by all. Warm, moist, dry, rarified and compressed atmospheres are agents that come in use to influence the physical conditions of our patients. According to each special case, we may select a climate where one or more of these agents come into action. By means of the pneumatic apparatus, we are not only able to produce at any time compressed, rarified, warmed and moistened air for our patients to inhale, but we can combine this mechanical with a local medicinal treatment, by passing the current of compressed air before it is inhaled through a flask partly filled with a medicated solution. At present, the following modes of treatment with the apparatus are practiced:

*Inhalations* of compressed air in cases of congestion of the lungs, stenosis and insufficiency of the mitral valve, chronic bronchitis, pleuritic residues and asthma. *Exhalation* in rarified air in all cases where the elasticity of the lungs and contractility of the chest are lost or impaired, emphysema, paralysis of the muscles of expiration, poisoning from carbonic oxide, etc. *Inhalation* of rarified air to exercise and strengthen the lungs and muscles of respiration in incipient consumption and predisposition to this disease. *Inhalation* of medicated compressed air in chronic bronchitis, etc, *Inhalation* of compressed air with exhalation in rarified air combined. This method has been found very useful in cases of emphysema complicated with chronic bronchitis. This method has all of the advantages of climato-therapy, without many of the discomforts often necessary to attain it.—Dr. A. E. Brune, of Sacramento, in *Pacific Med. & Surgical Journal*, August, 1880.

**New Method for Withdrawing Fluids from the Middle Ear.**—Dr. Charles Denison, of Denver, Col., states (in the Rocky Mountain Medical Review) that last winter, in the act of blowing his nose—having a cold at the time—catarrhal or other secretion was forced through the Eustachian tube into the middle ear, causing pain which was not relieved by the usual palliative measures. The experiment which gradually overcame the difficulty was this: Attaching the flat nozzle used with Pollitzer's air bag to the exhaust bottle of his aspirating apparatus, he first exhausted the air from the bottle, and then compressing the alæ of the nose, with the nozzle introduced so as to wholly exclude air from without, he closed his throat as in the act of swallowing, and kept it so, and at the same time directed the stop-cock to the vacuum bottle to be turned on. The effect of thus transferring the vacuum to the middle of the head was decided, seeming to draw everything inwards towards the pharynx and nasal passages, creating a temporary congestion of those parts. But the pain in the ear was perceptibly lessened by the experiment, and on its repetition finally wholly disappeared. He thinks that the effect was mechanical, and that a small portion of the fluid was probably drawn into the Eustachian tube at each operation. Since that time he has tried the experiment in practice as opportunity has offered, and mainly with results similar to those above mentioned. In one instance, he believes the stage of suppuration had set in, for he intended to puncture the membrana tympani the next day after this operation had been tried. But the patient did not return, and he found some time afterwards that improvement had continued from that time till she wholly recovered.—*Va. Medical Monthly.*

**Clover Tea for Cancer.**—This is not a new remedy, but it is one capable of doing good in some cases. At the request of a noble woman, who for years has suffered the agonies of cancer, and who has been greatly benefitted by this remedy, a writer in the Medical News wishes to re-awaken an interest in its use. She says: "The clover tea has done wonders for me. My appetite is now good, my general health greatly improved, and the wound is healing. For seven months I have had to take morphia, and its unpleasant effects had become great. My pain having so much diminished under the use of the clover tea, and my general health having gotten so much better, I determined to give up the morphia, and have gotten on comfortably without it. If my experience will save one poor suffering fellow creature a single pang such as I have suffered, I will thankfully bear my cross, and rejoice that through me a remedy has been found which will give relief, if not a cure, for cancer. The tea should be made as tea is made for table use, strained, and taken before meals and at bedtime, about a quart daily. The blossoms of red clover should be used.

A fluid extract has been made, of which the dose is a tablespoonful thrice daily.—*Va. Medical Monthly.*

**Tea as an Antidote to Opium.**—I have already published some remarks in the Dublin Medical Gazette, and also in the London Lancet, on the wonderful effects of tea as an antidote to opium. One of the first cases in which I had recourse to tea, was that of a lady who had taken a quantity of Batting's "black drop," so enormous that I

am almost afraid to mention the amount fearing that it may not be credited. However, as I had no reason to doubt the facts represented to me at the time, the quantity taken of the above named drug between 4 p. m. and 11 p. m. of a certain day was, as far as I can remember (having lost my note), ʒ xxviij, or ʒ ij every half hour for seven hours. But, let the dose be what it may, we have chiefly to do with its effects. At eleven o'clock she had a severe convulsion; at 11:30 or thereabouts I saw her, and found her in the following condition: Extremities perfectly cold, no pulse at the wrists, face pale, drawn, and cold, pupils contracted to a pin's point, and her respiration three in two minutes. To all appearances she was dying, but, being the wife of a medical man, and he absent, I sent for my colleague, Dr. Jackson, who arrived about midnight, and gave it as his opinion that she would not live ten minutes. While the messenger was absent for Dr. Jackson, I caused a strong infusion of green tea to be prepared, of which I administered half a pint as an injection. In twenty minutes, to my astonishment, as well as that of Dr. J., we could just discover the pulse at the wrist. There was the slightest tinge of red in the lips, while the respiration was *six in one minute* instead of *three in two minutes*. Encouraged by these wonderful results, another half pint was given at half-past twelve, after which she improved rapidly, so much so that at four o'clock in the morning she said to me, "please light the gas, I know your voice but cannot see you." The sun was shining brightly into the room at the time. I have been asked, "Why did you not give an emetic?" I answered, because I conceive no emetic would have had any effect on the paralyzed condition in which we may presume the stomach was after having received the enormous amount of opium above mentioned. I have also been asked why I did not use the stomach pump? Answer, because I do not always take a stomach pump with me when I go out at night, but chiefly because I could see no advantage to be derived from this instrument, seeing that a very large portion of the poison had already beed absorbed, and was now doing its fatal work. Moreover, I believe the attempt to introduce the tube in the prostrate state of the patient would have probably caused her death then and there. The remedy I think should always be administered by injection, as it is more likely to be quickly absorbed by a healthy bowel than a paralyzed stomach. Of course theine and caffeine would act probably quicker than the simple infusion, but the former remedies are not always at hand, while the tea is. The opium in this case was taken to relieve the pain of angina pectoris.—*Dr. Swell in Canada Medical Journal.*

**Points in the Surgery of the Urinary Organs which Every Practitioner Ought to Know.**—Teevan, in a paper recently read before the Harveian Society of London, called attention to the following points:

1. Retention of urine in children is always caused by stone, unless there is some mechanical obstruction to the escape of urine, such as a contracted meatus or foreskin.

2. Incontinence of urine, when diurnal as well as nocturnal, may be caused by a calculus impacted in the deep urethra. A stone would thus, in the one cace, give rise to retention, and in the other to incontinence, for the reason that when in the bladder and at the *meatus in-*

*ternus* it caused contraction of the sphincter which closely embraced it; advancing farther for half an inch down the canal, it acted as a gag preventing the contraction, and allowing the urine to dribble away by its side.

3. Incontinence of urine in boys may be caused by a congenitally contracted meatus, preventing the free escape of urine and setting up reflex action, resulting in the dribbling.

4. Dribbling of urine in men signifies retention, not incontinence. There is at first retention, until the bladder is overfilled, when gradually the obstruction is overcome by contraction of the bladder-walls, and then there is dribbling, the bladder still remaining distended.

5. When a catheter is introduced, if the urine is expelled with violence and pain, not only through the instrument but along its sides, between it and the urethral walls, there must be a calculus impacted in the deep urethra.

6. It is not possible to empty the bladder completely in every case, for the reason that it may be sacculated.

7. A gleet of more than six months standing implies an incipient stricture.

8. In cases of enlarged prostate, suspect a stone, for the reason that all the conditions necessary for its formation are present.

9. When a man, complaining of a frequent and painful micturition, is worse during the day than at night, he most likely has stone. In prostatic cases of frequent and painful micturition, the patient is much worse during the night. Calculus cases are most comfortable in bed; when they move about during the day they have pain from the movements being impressed on the stone.

10. When a man, complaining of frequent and painful micturition, is worse when riding on horseback, or in a vehicle, suspect stone, the reason being the same as just above.

11. See that the bladder of the woman in labor is empty before delivering the child.—*Western Lancet.*

**Action of Sugar on Calomel.**—From time to time notes have appeared in various journals, warning against the changes said to be produced in calomel by sugar and various other substances with which it is often prescribed; while many therapeutists advise that so long as calomel is being taken, no salted food or acid drink should be allowed. M. Verne has submitted these views to the test of experiment; he made various mixtures of calomel with common salt (in solution), beet-root and colonial sugar, solution of citric acid, etc.; these he examined at the end of periods varying from 3—15 days. Although at the end of these intervals the mixtures he experimented with showed to the eye sight traces of change, chemically he could find no evidence of the presence of corrosive sublimate, or of any soluble salt of mercury. He refers such accidents as have been reported chiefly to impurities in the drug; as supplied to the chemist, calomel is usually, to some extent, impure, and should always be washed with distilled water and alcohol. The danger of giving acid drinks with calomel is purely theoretical; it was proved that the latter, when exposed for fifteen days to the action of a 20 per cent. solution of citric acid, underwent not the slightest alteration. M. Verne concludes that the sub-chloride of mercury is a much more stable salt than is generally supposed; it is, in fact, more stable than corrosive sublimate, as solutions of the latter, when exposed to light and the influence of various organic substances, deposit calomel. Non-coagulated albumen, however, at the temperature of the human body, reduced calomel rapidly and easily, transforming it into a slightly soluble albuminate. Neither common salt nor sea salt, at 40° C., had any action on calomel, either when used alone or in the presence of albumen.—*Medical News and Abstract.*

**Hypodermic use of Quinine.**—Dr. Whittaker (in Lancet and Clinic) says: "I have, in practice, entirely discarded all vehicles except water, and rely solely upon heat to obtain a perfect solution. I have the druggist put into a test tube twenty grains of the bromide of quinine and add to it two drachms of water. The tube should be corked, not to preserve the substance, for it is still crystaline in this proportion, but for cleanliness. To use the drug, all that is necessary is to heat the tube over a gas flame, coal oil lamp, or other means of illumination. The tube should be held above the light, of course, and not in it, that it be not smoked, and hence rendered opaque. Two or three minutes suffice to reduce the quinine to limpid, crystaline fluid in the tube. Thence it is poured, then, in sufficient quantity into a teaspoon, previously warmed by holding one minute over the flame, and thence from the spoon it is taken up into the syringe, warmed also in the same way, and is ready for use, which must be immediate. It may be injected anywhere, but always *under* and never *into* the skin. The ordinary syringe contains half a drachm, and this introduces about five grains at a time.

I have never known a patient to object to the introduction of the needle for the injection of ten or fifteen grains, if need be. The whole operation, no previous preparation being necessary, occupies about five minutes time, not a tithe of that often consumed in irrelevant conversation.

**Erythoxylon Coca on the Opium and Alcohol Habits.**—Dr. Bentley, in Therapeutic Gazette, reports the following cases:

Miss M., a sprightly, intelligent blonde, æt. 30. I had been acquainted with her for 15 years, and for several years of our early acquaintance, when the family lived near me, was her father's family physician. In June, 1878, I accidentally met her on a train. Eight years previous she had a protracted attack of pneumonia, during which she contracted the habit of using morphine, which she still retained. These facts were well known to me, so I took occasion to ask her if she had left it off. She replied in the negative, and told me that she then required 10 grs. twice a day. She had tried nostrums and had visited some advertising quack in another State, to no purpose. I then suggested the erythoxylon, fully explaining its action. The result was I prescribed a pound for her. I received a note from her when she had used this. She was much encouraged, and had ordered two pounds more. This quantity completed the cure. I saw her recently, when she assured me that she had no desire for morphine.

Case 2. July, 1878, was called to a case some 15 miles distant, and nearly all the way "in the hills." I was quite astonished to find about one-tenth of an acre in poppies. On enquiry, the lady of the house, a widow, æt. 40, told me that she was an "opium eater," to use her expression, and that she used her own opium. From what I could learn, she used about half a pound of the drug a year. I persuaded her to give up the habit. She declared that she could not. She agreed, however, to try, so I sent her ½ lb. fld. extr. coca to begin with. When used, she sent for half the quantity, stating that she thought it would complete the cure. I sent her a half pound. She sent me her opium crop that winter, with the message that the medicine had cured her.

Case 3. An aged lady, æt. 72. She had been addicted to the habit for 35 years. I persuaded her to try the coca. She was then taking ½ drachm doses of opium three times a day. She said she felt sure she would be unable to leave off the opium, but as she was wealthy and extravagant, she readily consented to try the coca. She procured two pounds on my prescription, and began its use. The progress of the case is rather amusing. She uses one for a time, and then resorts to the other. Sometimes she does not taste opium for a fortnight, using the coca during the time, then she returns to her opium, and thus she alternates. Her doses of opium have been reduced, to as little, I think, as 10 grs., and her general health has greatly improved. Before using the coca, she was a great sufferer from duodenal dyspepsia. She has so far improved, in this respect, that she rarely suffers in this particular, and it is a wonder, too, for she is a ravenous feeder, and as fond of "cake and pastry" as a child, and, withal, uses a great deal of wine.

Case 4. An unmarried man, æt. 27. Contracted the "opium habit" five years previous to using the coca. Had become a great slave to morphine. June, 1879, I put him upon coca. He ordered three pounds at the beginning. In October following, I met him, and he assured me that he was entirely relieved of the habit, and had one pound of his medicine left.

**Treatment of Scarlet Fever by Warm Baths.**—The following communication from W. Vawdrey Lush, M.D., physician to the Dorset County Hospital, appeared in The Lancet, Aug. 14, 1880:

In Dec., 1869, while we were experimenting with a very severe epidemic of scarlet fever, there appeared in The Lancet a reprint of a letter by Dr. Charles T. Thompson, strongly advoceting the use of warm baths in this disease, and stating that he had pursued the practice for fifteen years, and had never lost a patient.

In consequence of this communication, I commenced this practice ten years ago, and have followed it from that time to the present. At first I ordered the patient to have three warm baths daily, to be kept in from three to five minutes, rapidly dried, wrapped in a blanket, and returned to bed. As the disease subsides, I reduced the baths to two or only one daily. I find that—1st, it brings out the rash; 2d, reduces the temperature; 3d, soothes the patient; and when this treatment has been adopted at the outset, I have as yet not lost a single patient.

In one case the warm bath was objected to till the child had been ill several days, and this case, and this alone, proved fatal.

My friend, Dr. Alfred Hollis, of Freshwater, has told me of the great comfort he himself experienced from warm bathing when suffering from the disease; and, of course, in the treatment neither medicine proper nor good nursing is precluded.

Some of my readers may recollect a case of small-pox published by the late Dr. Stokes, of Dublin, where the warm bath proved singularly beneficial, and who doubted not that the mortality in small-pox hospitals would be greatly diminished by the use of the bath. The case I refer to was that of a medical student, in which "the postulation was almost universally confluent; the purulent matter highly putrescent; the hemorrhagic state developed, the body one universal ulcerous sore, and the blackness of the worst purpura developed; the odor of an intensely pungent and offensive character, which seemed to pass through the bystander like a sword. Stimulants alone, freely and constantly employed, seemed to preserve the patient alive. The pulse was rapid, weak and intermittent, and for several days life was despaired of. At this juncture, Dr. Stokes happened to describe the case to his colleague, Mr. Smyly, who suggested the trial of the warm bath. Pillows were adjusted in one, the patient placed in it, and the effect was instantaneous and marvelous. The delirium immediately ceased. The patient exclaimed, "I am in heaven! I am in heaven! Why didn't you do this before?" He was kept at least seven hours in the bath, brandy being freely administered, and removed to bed. The bath was repeated the next day, after which he fell for the first time into a tranquil slumber. From this time recovery was progressive."

This may seem a digression; but the treatment of another of the exanthemata by similar means is not inapposite.

My ten years added to Dr. Thompson's fifteen make twenty-five years' experience of a treatment which I can confidently and heartily recommend. *Mich. Med. News.*

**Hot Rectal Douche.**—Dr. Chadwick, of Boston, in a paper before the American Gynecological Society, "Recommended the use of hot rectal douche for arresting diarrhœa and abdominal pain, and for pelvic inflammations of all kinds. Hot water thus brought into the abdominal cavity will encircle the whole mass of pelvic organs and produce a wonderful effect upon inflamed organs. After citing a number of cases, the Doctor remarked: "The water should be as hot as the hand can endure; while using the douche pass the finger into the vagina with the palm backward. The minute you begin to feel the lower pouch filling up you must pause a moment, without a withdrawal of the nozzle. In this wise from one to four pints of water may be introduced without exciting immediate action. The patient must be quiet for about a half hour. It is not wise for the patient to resist the expulsive efforts of the muscles. The peristaltic action is very variable. Sometimes it will be set up very soon, and sometimes not for hours. I am unable to say how far up the water usually passes, but I am satisfied it passes through the large intestine to the valve. Retrostalsis, I am satisfied, does occur under some circumstances. I recommend its use two or three times per day for a week or so, and then sometimes to be discontinued for a week. I recommend the douche principally for an inflammatory condition of the large intestines or the rectum, secondarily, a condition of the pelvic organs characterized by painful defecation or burning sensations about the ovaries."

**Ulceration of the Bowel following the Injection of a Pile with Carbolic Acid.**—Mrs. J. H. W., from an interior town in Kentucky, consulted me at the instance of her family physician, for rectal trouble, in July last. She gave this as the history of the affection: For several years she had suffered with what she had supposed to be internal piles. The first few months she lost blood both at stool and in the interval. Nothing at that time protruded from the bowel. Later on, however, the bleeding seemed to check, and a protrusion of a tumor would take place while at stool. After action the protruding part would retract of itself. Very little pain was experienced during the entire time, but for the inconvenience she sought the advice of an advertising physician. He diagnosed the case as one of internal piles, and recommended an operation. An injection of the tumors was practiced, consisting, it was inferred, of carbolic acid, of what strength is not known. The piles sloughed, and the woman believed herself to be cured, but after her return home pain manifested itself at stool, which would continue for hours after. The condition continued to grow worse until the time of her application to me. Upon an examination, an ulcer was found above the external sphincter muscle, and bordering upon the internal. It was about one-half inch in diameter, and looked very angry. It was caused, as could be very easily seen, by the sloughing of the tissues from the injection into the pile. She was at once put upon the proper treatment, but it was some weeks before she could be pronounced well. This is but one of a number of cases of ulceration following the injection of carbolic acid into piles, that has been seen by the writer, and is cited to show that sloughing is one of the immediate dangers to be anticipated in the carbolic treatment of piles.—*Louisville Med. Herald*, Nov.

**Miss Adelaide Neilson—Cause of Her Death.**—Dr. W. E. Johnston, of Paris, who frequently attended Miss Neilson during attacks of illness, states that the disease from which she suffered was principally gastralgia, dependent quite as much on moral causes as on errors in diet. In her last fatal attack, during a most violent recurrence of pain, she suddenly ceased to complain, went into a state of syncope, and died in the syncope. The post mortem examination made the next day disclosed the extraordinary fact, one of the rarest in the history of medicine, that in her writhings she had ruptured a varicose vein in the left Fallopian tube, and had died from internal hæemorrhage. Two quarts and a half of blood were found in the peritoneal cavity, and the ruptured vein presented an orifice of from four to five millimetres in diameter.—*Maryland Med. Jour.*

**Aihun.**—Aihun is called, in Brazil, a disease peculiar to the negro race of that country, which affects the small toes of the feet.

It is, according to the opinion of Dr. Guimaraes, a *sui generis gangrena*—a steady, slow, mysterious work of decomposition, only limited to the small toes of the feet, probably due to the lack of nourishment of these parts, that causes a retrograde metamorphosis in them, and gradually their final elimination from the body, without giving much trouble, at least in the commencement, to the general system.

The disease never appears among the white people. The first who called the attention of the medical world in Brazil was Dr. Moncorco de Figueiredo. In a paper read before the Medical Academy of Rio Janeiro, in 1874, he related a case operated by him, and presented, at the same time, the pathological specimens.

Farther investigations were since pursued by other distinguished practitioners of that empire, such as Drs. Silva Lima, and Domingos de Almeida Martins Costa.

Later, Dr. Guimaraes has discovered an exception to the rule—that is, he had a case in which, not only the small toes were affected, but also the fourth.

The proximate cause of these strange phenomena is supposed to be in the contracture of the arteries that nourish the toes.

Not much is known with regard to the treatment of *aihun.—N. O. Med. Jour.*

**The Two Bogus Colleges Finally Wiped Out,**—The American University of Philadelphia, and the Eclectic Medical College of Pennsylvania, known to the community as Buchanan's Colleges, were, on September 30th, finally wiped out of existence. J. Howard Gendell, who represented the Commonwealth of Pennsylvania, filed his replication to the answers, put in by the colleges some time ago, to the quo warranto proceedings against them. The answers of these two corporations were, that they had a right to exercise their franchises by acts of the Legislature. Mr. Gendell's replication set out substantially that the colleges forfeited their rights under those acts, because they conferred degrees upon persons not possessing the qualifications prescribed in their charter; by selling diplomas; by granting degrees for doctor of medicine and antedating the diplomas so as to make it appear that the party had a right to practice medicine; and finally, by issuing diplomas with forged signatures After the replication was filed, the counsel for Dr. Buchanan confessed judgment of ouster, and filed, as part of the record, a letter from Dr. Buchanan instructing him to do so.—*Med. and Surg. Rep.*, Oct. 9.

**Parotitis as a Complication of Ovariotomy.**—R. Moricke says that the not very uncommon concurrence of parotitis and inflammation of the testicle has its analogue in the female sex, certain cases having been recorded where parotitis followed or became intercharged with disease of the female sexual organs. Among two hundred ovariotomies reported by Schroder, parotitis was observed in five cases; only one of these could be referred to infectious disease, consequently there could be no question of metastasis. The enlargement began in one case on the fourth day, in the other cases from the sixth to the seventh day. In these cases suppuration of the parotid gland occurred. One patient died of this affection, so that in debilitated individuals it may be regarded as a serious complication of avariotomy. The relationship of the two affections is difficult to understand.—*Phil. Med. Times.*

**Painless Operation for Ingrowing Nails.**—Dr. J. H. Converse, in American Medical Journal, says: "I will give this for the benefit of your readers, as perhaps it will be new to some of them. It consists of wedging cotton under the free margin of the nail, placing over it a piece of adhesive plaster with a hole cut into it the size and shape of nail to be removed; then moisten the end of a pencil of caustic silver and apply it to part to be removed, taking care not to touch any other portion. The next day the nail will have assumed a black or brown appearance. Upon raising the nail it will be found to have become separated from the sub-adjacent tissue, and all that is required to complete the cure is to clip off the dead portion."

[We think it doubtful whether the cauterized portion can be clipped as early as the next day, as above stated, but publish it that it may be tested by our readers.—ED. REC.]

**Muriate of Ammonia in Intermittents.**—Dr. Smythe, in Michigan Medical News, in cases where a troublesome rash results from the use of quinine, says: "To avoid the liability of causing this very disagreeable affection, in all the subjects, who are known to be liable to be attacked by this peculiar rash when suffering with intermittents, (I have several such patients, my son being one), I have for many years substituted the hydrochlorate of ammonia, in powder, with the most happy and successful results. Fifteen grains every two hours, commencing eight or ten hours before the return of the paroxysm, four or five doses generally answering for the time, to be repeated for two or three days, thereafter in the same way."

**Arsenic in Consumption.**—In the Medical Press and Circular, Dr. Wm. A. Pearse says the success of the following combination in many cases of consumption has been so great that he feels it a duty to bring it before the profession.

℞ Liqu. arsen. hydrochlor, *m*lxiv., quinia sulph. grs. viij., acid hydrochlor dil. ʒiij., syrup aurant. ℥j., infus. chirettæ ad. ℥viij. M.

This mixture equals sixteen doses, of which one was taken three times a day, after meals. In many cases ten minims of sea water were added to each dose; in others two grains of sulphate of manganese. The patients were directed to continue the medicine during six weeks, then to allow an interval of a week, and again to resume treatment.—*Medical Journal.*

**Fasting or Starvation.**—Mr. George Fleming, F.R.C.V.S., in the Veterinary Journal for September, referring to the supposed fasting experiment of Dr. Tanner, says that a similar cruel attempt was made with a number of horses in Paris in the spring of 1876. There was, indeed, this difference between the two cases, that the fast was forced upon the poor quadrupeds without their consent, and that there was a pretense of utility about the French experiment. The aim, as it was stated at the time, was to discover how long horses could go without food in the event of the scarcity which accompanies a state of siege. The following results were obtained from the inhuman experiment:

1. It was proved beyond all doubt that a horse can hold out for twenty-five days without any solid nourishment, provided it is supplied with sufficient and good drinking water.
2. A horse can barely hold out five days without water.
3. If a horse is well-fed for ten days, but insufficiently provided with water throughout the same period, it will not outlive the eleventh day.

One horse, from which water had been entirely withheld for three days, drank on the fourth day sixty liters of water within three minutes. A horse which received no solid nourishment for twelve days was nevertheless in a condition on the twelfth day of its fast to draw a load of two hundred and seventy-nine kilos.—*Louisville Medical News.*

**The Topical Use of Ergot.**—The value of the local use of ergot is receiving endorsements from various quarters. It is particularly applicable in catarrhal diseases of the eye and throat. In chronic conjunctivitis it may be employed in the strength of gr. x. of the extract to ℥ j. of water, a little glycerine being added to preserve the drug. In chronic pharyngitis, when the secretion is not very great, it makes an excellent ingredient of a gargle, or it may be used with tincture of iodine and applied with a probang. In cases of nasal catarrh, it may be applied by means of gelatine bougies. In using the ergot it should generally be combined with glycerine, as that agent both preserves it from decomposition, and keeps it longer in contact with the diseased surface.—*Medical Press and Circular.*

**Quinine as an Anti-Abortive.**—While many claim that quinine is capable of exciting uterine pains, we find Dr. Campbell, in American Gynecological Gazette, takes "the ground that the liberal use of quinine in malarial regions is absolutely essential to prevent abortion, and was sustained in his opinion by most of the debaters who took part in the discussion."

**Summer Complaint.**—Essence of camphor is recommended for summer diarrhœa of children. Five drops on a piece of sugar every ten minutes or a quarter of an hour, according to the severity of the symptoms, will nearly always stop it.

**Beri-Beri.**—Beri-beri is a constitutional disease of an infectious nature, whose etiology is unknown; assuming a mixed condition of paralysis and œdema, characterized by dyspnœa; disorder of the organs of digestion, of respiration, of circulation, and principally of the nervous system. The disease properly belongs to the tropical regions, where it prevails endemically or epidemically.

**Treatment of Puerperal Fever.**—Dr. Bell finds that no remedy is so effectual in purifying the system in cases of puerperal fever as the Edinburgh preparation of the tincture of the muriate of iron, when given regularly in full doses frequently repeated (*e. g.*, thirty drops every two hours.) The great error in the employment of this medicine is the timidity shown in giving it in sufficient doses; in consequence its good effects have been questioned in other diseases of a zymotic character, such as erysipelas, diphtheria, and scarlet fever. It has a remarkable effect in moderating the pulse and diminishing the secretion of pus. Dr. Bell thinks it right, however, to warn the practitioner against trusting to a new preparation of iron called the tinct. ferri perchloridi, which differs from the tinct. ferri muriatis in its formation, its medicinal effects, and in its analysis.—*The Edinburgh Medical Journal.*

